# Dapagliflozin effect on endothelial dysfunction in diabetic patients with atherosclerotic disease: a randomized active-controlled trial

**DOI:** 10.1186/s12933-021-01264-z

**Published:** 2021-03-26

**Authors:** Andrei C. Sposito, Ikaro Breder, Alexandre A. S. Soares, Sheila T. Kimura-Medorima, Daniel B. Munhoz, Riobaldo M. R. Cintra, Isabella Bonilha, Daniela C. Oliveira, Jessica Cunha Breder, Pamela Cavalcante, Camila Moreira, Filipe A. Moura, Jose Carlos de Lima-Junior, Helison R. P. do Carmo, Joaquim Barreto, Wilson Nadruz, Luiz Sergio F. Carvalho, Thiago Quinaglia, ADDENDA-BHS2 trial investigators

**Affiliations:** Aterosclerose and Vascular Biology Laboratory (Aterolab), Cardiology Division, State University of Campinas Medical School, Rua Tessalia Vieira de Camargo 126, Cidade Universitaria Zeferino Vaz, Campinas, SP 13084-971 Brazil

**Keywords:** SGLT2, Endothelial function, Type 2 diabetes, Nitric oxide

## Abstract

**Background:**

The glucose-lowering independent effect of sodium glucose cotransporter-2 inhibitors (SGLT2i) on arterial wall function has not yet been clarified. This study aims to assess whether SGLT2i treatment can attenuate endothelial dysfunction related to type 2 diabetes mellitus (T2D) compared with glucose-lowering equivalent therapy.

**Methods:**

In a prospective, open-label, single-center, randomized clinical trial, 98 patients with T2DM and carotid intima-media thickness above the 75th percentile were randomized 1:1 to 12 weeks of therapy with dapagliflozin or glibenclamide in addition to metformin in glucose-lowering equivalent regimens. The coprimary endpoints were 1-min flow-mediated dilation (FMD) at rest and 1-min FMD after 15 min of ischemia followed by 15 min of reperfusion time (I/R).

**Results:**

Ninety-seven patients (61% males, 57 ± 7 years) completed the study. The median HbA1c decreased by − 0.8 (0.7)% and -0.7 (0.95)% following dapagliflozin and glibenclamide, respectively. The first coprimary endpoint, i.e., rest FMD changed by + 3.3(8.2)% and − 1.2(7.5)% for the dapagliflozin and glibenclamide arms, respectively (p = 0.0001). Differences between study arms in the second coprimary endpoint were not significant. Plasma nitrite 1 min after rest FMD was higher for dapagliflozin [308(220) nmol/L] than for glibenclamide (258[110] nmol/L; p = 0.028). The resistive indices at 1 min [0.90 (0.11) vs. 0.93 (0.07); p = 0.03] and 5 min [0.93 (0.07) vs. 0.95 (0.05); p = 0.02] were higher for the glibenclamide group than for the dapagliflozin group. Plasma biomarkers for inflammation and oxidative stress did not differ between the treatments.

**Conclusions:**

Dapagliflozin improved micro- and macrovascular endothelial function compared to glibenclamide, regardless of glycemic control in patients with T2DM and subclinical carotid atherosclerotic disease.

**Supplementary Information:**

The online version contains supplementary material available at 10.1186/s12933-021-01264-z.

## Highlights

- Sodium-glucose cotransporter-2 inhibitors (SGLT2i) may reduce the risk of myocardial infarction in individuals in secondary prevention but not in those with uneventful clinical history
- Subclinical atherosclerotic disease, including carotid disease, is a strong marker of cardiovascular risk
- Endothelial dysfunction is a potent marker of cardiovascular risk, and its attenuation is an indicator of potential clinical benefit.
- A 12-week course of dapagliflozin improves vasomotor function in the macro- and microcirculation of patients with T2DM and carotid atherosclerotic disease when compared to a 12-week glibenclamide course in the setting of equivalent glycemic control.
- Individuals with subclinical atherosclerosis can potentially benefit from treatment with SGLT2i to achieve cardiovascular benefit.

## Background

Sodium-glucose cotransporter-2 inhibitors (SGLT2i) have been investigated in several large studies [1–3]. Meta-analysis based on these clinical trials suggested that SGLT2i reduce the risk of myocardial infarction in individuals with prior manifestation of coronary events but not in those with uneventful clinical history [4]. This set of evidence generated, as a clinical guideline, an expectation of vascular benefit with the use of SGLT2i only in individuals with a prior history of cardiovascular disease (CVD) [5, 6]. Nevertheless, these studies were not designed to investigate early signs of benefit in the arterial wall, such as a change in endothelial function and were not long enough to verify attenuation in the incidence of atherosclerotic disease except in these individuals at very high cardiovascular risk.

There is great heterogeneity in cardiovascular risk among individuals with type 2 diabetes mellitus (T2DM). In general, the detection of subclinical atherosclerosis is a strong predictor of cardiovascular risk because it yields an equivalent risk status to prior CVD [7]. Among the strategies to detect subclinical disease, the investigation of carotid atherosclerosis has been shown to be useful for predicting cardiovascular events in individuals with or without previous CVD [8]. Therefore, detecting carotid atherosclerotic disease in individuals with T2DM may identify a subgroup that would achieve vascular benefit with SGLT2i regardless of the preexistence of manifested CVD. To the best of our knowledge, there are no data supporting this hypothesis to date.

Impaired endothelial function is one of the first disorders detected in atherogenesis and can be modified early by prevention therapies [9]. For this reason, changes in endothelial function have been used as surrogate endpoints, particularly in short-term studies [10]. Flow-mediated dilation (FMD) of the brachial artery is a preferred method for assessing endothelial function owing to its noninvasiveness, close correlation to coronary endothelial function [11] and association with the incidence of long-term coronary events [12]. Moreover, concomitant measurement of endothelium-derived biomarkers improves the accuracy of endothelial function evaluation.

Endothelial function is often impaired in patients with T2DM and exacerbated by ischemia and reperfusion (I/R) injury [13]. Imbalances between constrictive and relaxing endothelium-derived factors such as nitric oxide (NO) and endothelin-1 reduce artery blood flow and aggravate ischemia [14]. Hence, improving endothelial function may also attenuate I/R injury in T2DM patients. Data from animal and cell models suggest that SGLT2i does not increase the eNOS transcription rate [15] but may change its phosphorylation, thereby increasing NO production [16]. Since NO generation is closely related to vasomotion and protection from I/R injury [17], SGLT2i can theoretically improve both macrovascular and microvascular function at rest and increase residual endothelial function after a period of I/R stimulus. The present trial was based on the hypothesis that the administration of SGLT2i in patients with T2DM and atherosclerotic disease may (i) mitigate endothelial dysfunction and (ii) increase endothelial resilience to I/R injury regardless of its glucose-lowering effect. We carefully considered the importance of equivalent glycemic control as well as the presence of structural and functional changes in the artery wall as requirements for designing our study.

## Methods

The Assessment of Dapagliflozin Effect on Diabetic Endothelial Dysfunction of Brachial Artery-Brazilian Heart Study 2 (ADDENDA-BHS2) was a randomized, open-label, single-center study comparing equipotent glucose-lowering regimens of glibenclamide combined with metformin versus dapagliflozin combined with metformin in patients with T2DM and subclinical carotid atherosclerotic disease. Full details regarding the study selection criteria, procedures, and assessment of brachial artery function are published elsewhere [18] and described in depth in the Additional file 1. Briefly, eligible patients were 40–70 years of age. Inclusion criteria included a glycated hemoglobin (HbA1c) level between 7 and 9%, the use of ≤ two oral hypoglycemic agents and the presence of atherosclerotic disease identified by carotid ultrasound. Prior manifestation of CVD (myocardial infarction or coronary angiography with at least one coronary artery with ≥ 70% stenosis) was considered an alternative criterion for atherosclerotic disease. Exclusion criteria included atrial fibrillation, chronic kidney disease, hepatic dysfunction, uncontrolled hypertension, acute coronary or cerebrovascular disease within 6 months before enrollment, plasma triglyceride levels > 500 mg/dL, clinical suspicion of volume depletion, insulin use, and pregnancy or reproductive age in female patients.

Eligible patients underwent an initial 16-week run-in phase (Additional file 1: Figure S1) where they were administered ≥ 1.5 g/day extended-release metformin and 25–100 mg/day losartan. Patients who remained eligible after the initial medication adjustment period were randomly assigned to a 12-week treatment phase at a 1:1 ratio to dapagliflozin (10 mg/day) or glibenclamide (5 mg/day) on top of their ongoing metformin use. The objective of this run-in phase was to obtain equivalent control of blood glucose and blood pressure (BP) before randomization.

Statins were used by 45% and 43% of patients prior to randomization, respectively (Table 1). We did not introduce statins during the run-in period to avoid inaccuracy in assessing the effect of SGLT2i. This decision was based on previous studies that demonstrated a broadly heterogeneous change in FMD in individuals with diabetes after statin therapy [19]. Patients were maintained in the run-in medication regimens without any changes throughout the study period except for the addition of randomized therapies.

**Table 1:** Baseline characteristics

	Dapagliflozin	Glibenclamide	p
n	48	49	
Gender male, %	60	61	1.0
Age, years	57 ± 7	58 ± 7	0.4
T2DM duration, years	9 ± 7	10 ± 7	0.28
Distal polyneuropathy, %	42	41	1.0
Hypertension, %	83	76	0.45
Prior smoking habit, %	44	38	0.5
Sedentarity, %	54	65	0.3
Heart rate, bpm	71 ± 10	73 ± 10	0.28
Office systolic blood pressure, mm Hg	136 ± 12	138 ± 15	0.67
Office Diastolic blood pressure, mm Hg	81 ± 9	83 ± 9	0.4
24-h Systolic blood pressure, mm Hg	124 ± 10	127 ± 11	0.1
24-h Diastolic blood pressure, mm Hg	73 ± 20	72 ± 21	0.88
Body mass index, kg/m^2^	31 ± 4	30 ± 5	0.76
Waist circumference, cm	105 ± 11	103 ± 10	0.32
Fasting blood glucose, mg/dL	174 ± 43	174 ± 57	0.99
HbA1c, %	7.9 ± 0.9	7.9 ± 0.9	0.74
Plasma insulin, mUI/mL	14.3 ± 6.7	15.5 ± 13.6	0.66
HOMA-IR	6.2 ± 3.3	6.2 ± 3.9	0.66
LDL-cholesterol, mg/dL	93 ± 30	96 ± 35	0.76
HDL-cholesterol, mg/dL	41 ± 11	41 ± 10	0.62
Triglycerides, mg/dL	179 ± 112	174 ± 94	0.76
Glomerular filtration rate, mL/min	95 ± 20	92 ± 19	0.39
Urinary albumin/creatinine ratio, mg/g	0.2 ± 0.2	0.3 ± 0.3	0.29
Carotid IMT, mm	1.0 ± 0.2	1.0 ± 0.1	0.15
Carotid plaque, %	79	74	0.6
Nitrate levels during FMD, nmol/L			
At baseline	36.0 (22.5)	30.0 (21.0)	0.444
At 1-min	35.4 (18)	29.2 (14.3)	0.323
At 5-min	31.9 (19.2)	28.7 (14.0)	0.273
AUC during the 5-min course of FMD	69.9 (34)	59.7 (31.9)	0.436
Nitrite levels during FMD, nmol/L			
At baseline	25.9 (15.0)	24.2 (17.0)	0.692
At 1-min	24.1 (13.0)	25.0 (10.0)	0.231
At 5-min	27.1 (17.0)	25.6 (16.0)	0.420
AUC during the 5-min course of FMD	50.0 (25)	52.0 (22.0)	0.111
Whole blood viscosity, cPs	5.65 (0.72)	5.49 (0.66)	0.155
Rest FMD at 1 min, %	1.57 (5.29)	1.23 (3.98)	0.918
Post I/R FMD at 1 min, %	1.45 (5.54)	1.03 (4.87)	0.761
AUC of arterial diameter during the 5-min course of the Rest FMD, mm	29.17 ± 4.92	28.65 ± 4.72	0.601
Peak of systolic velocity, cm/sec			
At baseline	65.6 (34.5)	65.1 (20.9)	0.655
At 1-min	83.2 (30.9)	84.2 (27.9)	0.619
At 5-min	73.6 (29.0)	73.0 (15.6)	0.971
End diastolic velocity, cm/sec			
At baseline	5.11 (1.7)	4.93 (3.3)	0.464
At 1-min	8.4 (6.8)	6.8 (8.0)	0.718
At 5-min	4.1 (2.3)	4.0 (2.1)	0.789
Resistive index			
At baseline	0.92 (0.04)	0.93 (0.04)	0.084
At 1-min	0.90 (0.08)	0.91 (0.09)	0.36
	0.95 (0.03)	0.94 (0.03)	0.761

Patient blood samples, office BP measurements, and 24-h ambulatory BP monitoring (24-h ABPM) were obtained at randomization and after 12 weeks of treatment. Brachial artery FMD at rest and after I/R was obtained in a systematic method as recommended by the Expert Consensus for the assessment of FMD [20] at randomization and after 12 weeks of treatment. FMD recordings were obtained by trained physicians and were blindly analyzed at the end of the trial. Further details on how the measurements were obtained can be found in full detail in the Additional file 1.

The study is reported according to the recommendations of the SPIRIT statement [21]. The ADDENDA-BHS2 trial was registered at Clinicaltrials.gov on 29 September 2016 under No. NCT 02,919,345. The screening started on July 1, 2017, and the first trial randomization occurred on July 27, 2017. The post-trial evaluation of the last patient enrolled in the study was performed on December 12th, 2028.

### Endpoints

The study was designed to determine whether there is a difference in endothelial function between treatment with dapagliflozin and glibenclamide. The two primary endpoints were (i) the change in rest FMD at 1 min measured at randomization and after 12 weeks of treatment and ii) the change in post-I/R FMD at 1 min measured at randomization and after 12 weeks of treatment. The detailed methods in which FMD was obtained in each situation are described in the Additional file 1. Prespecified secondary parameters included the area under the curve (AUC) of the diameter vs. time scatterplot and the derived parameters of blood flow velocity, arterial diameter and shear rate, plasma viscosity, and vascular and inflammatory marker activity levels.

### Statistical analysis

Sample size calculations have been published elsewhere [18]. Briefly, the two study endpoints equally split an alpha level at the bilateral α = 0.025. If one of them proved significant, the alpha value could then be recycled to test the secondary endpoints based on the index primary endpoint at the bilateral α = 0.05. Baseline continuous and categorical data were compared by Student’s *t* or Wilcoxon-Mann–Whitney *U* tests and a two-tailed Fisher’s exact test, respectively. Data are presented as the mean ± standard deviation (SD) for normally distributed data and medians and interquartile ranges (IQRs) for non-normally distributed data. The analyses were conducted according to the intention-to-treat principle. Changes from baseline were compared between treatments for each primary endpoint by analysis of covariance (ANCOVA) adjusted for baseline values. Prerequisites for the ANCOVA models (linearity, distribution normality and equal variance) were checked using histograms, normal probability graphs and residual dispersion. Exploratory intragroup comparisons of pretreatment vs. posttreatment values were performed by a related-samples Wilcoxon signed rank test or Student’s *t-test* paired for variables with non-normal or normal distribution, respectively. Analyses were performed using SPSS v.22 for Macintosh (IBM Corp., Armonk, NY, USA).

## Results

### Patients’ selection

The CONSORT flow diagram describing all enrollment processes is shown in the Additional file 1: Fig. S2. All patients were above the 75th percentile for patient age, sex, and race based on their distribution in the Brazilian population. Eight patients also had been previously diagnosed with CVD and were evenly distributed between the two study arms.

### Run-in phase

Before randomization, patients remained in the run-in phase to adjust medical treatments for a median of 68 (62) days for those who were lately allocated into the dapagliflozin arm and 89 (91) days for those who were allocated into the glibenclamide arm (p = 0.13). At the end of the run-in, the final metformin dose was similar for patients in the dapagliflozin arm (median 1700 mg/day [50]) and glibenclamide arm (median 1750 mg/day [50]; p = 0.75). The mean final dose of losartan was the same for both groups (median 100 mg/day [50]). To ensure stable clinical control, after reaching the prespecified therapeutic targets, patients remained without new changes in their therapeutic regimens for at least 45 days. After this period, only those patients who remained in the therapeutic targets were randomized.

### After randomization

As one patient requested withdrawal before concluding the study assessments, the analyses were performed on data from 49 patients in the glibenclamide arm and 48 patients in the dapagliflozin arm (Table 1). There were no significant differences in baseline characteristics between the study arms. No patient was an active smoker, and those who were previously smokers (~ 40%) stopped smoking 19 ± 9 years ago.

There was no difference between groups in terms of HbA1c either at randomization or at week 12. Median HbA1c reductions of − 0.8 (0.7)% and − 0.7 (0.95)% for the dapagliflozin and glibenclamide groups, respectively. Similar results were obtained for the plasma glucose levels (median reductions of [− 31(46) mg/dL] and [− 34(54) mg/dL] for the glibenclamide and dapagliflozin groups, respectively).

At 12 weeks, the systolic BP was lower (130 ± 17 vs. 137 ± 16 mm Hg; p = 0·035), and the diastolic BP tended to be lower (78 ± 10 vs. 81 ± 9 mm Hg; p = 0·054) in the dapagliflozin group than in the glibenclamide arm. In contrast, the mean systolic BP during the 24-h ABPM was similar in both arms at randomization and at the end of the trial (126 ± 13 vs. 126 ± 12 mmHg; p = 0.98 for dapagliflozin and glibenclamide arms, respectively). Similarly, the mean diastolic BP obtained during 24-h ABPM was equivalent in both arms at the beginning and the end of treatments (77 ± 8 vs. 77 ± 8 mmHg; p = 0.78 for the dapagliflozin and glibenclamide arms, respectively). The average daytime systolic BP (baseline: 126 ± 10 vs. 128 ± 10 mmHg; 12-week: 127 ± 13 vs. 127 ± 12 mmHg; p > 0.05 for both) and diastolic BP (baseline: 77 ± 9 vs. 77 ± 8 mmHg; 12-week: 77 ± 9 vs. 77 ± 8 mmHg; p > 0.05 for both) were also equivalent in both the dapagliflozin and glibenclamide arms.

Whole-blood viscosity increased [0.2 (0.43) cPs; p = 0.001] after dapagliflozin treatment but not after glibenclamide treatment [0.007 (0.43) cPs; p = 0.624]. Patients experienced a weight loss of 1.7 (2.3) kg with dapagliflozin and a weight gain of 1.1 (2.5) kg with glibenclamide (p < 0.001).

### FMD parameters

**Fig. 1:**
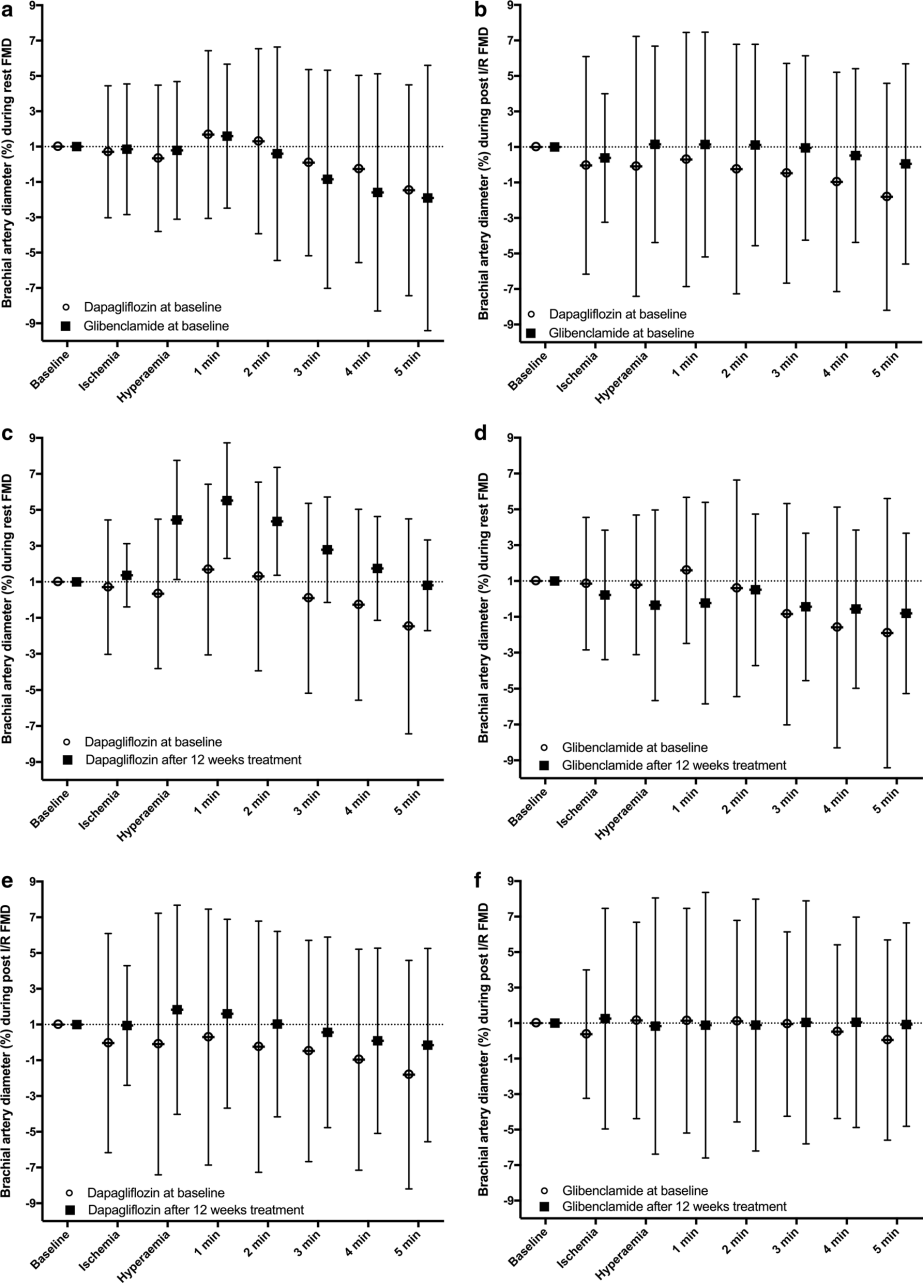
Time course of FMD measured at rest and after I/R injury in patients administered dapagliflozin (circle) or glibenclamide (square). Graphs A and B plot resting (**a**) and post I/R (**b**) curves in both study arms during the prerandomization phase. Graphs **c** and **d** show resting FMD curves for the dapagliflozin (**c**) and glibenclamide (**d**) arms before and after 12 weeks of treatment. Graphs **e** and **f** show post-I/R FMD curves for the dapagliflozin (**e**) and glibenclamide (**f**) arms before and after 12 weeks of treatment

The posttreatment change in rest FMD at 1 min (the first coprimary endpoint) was significantly different between dapagliflozin and glibenclamide (Fig. 1c and d; Table 2). For the dapagliflozin group, there was a median 3.3% increase between baseline and 12 weeks, whereas a median 1.2% decrease was measured for the glibenclamide arm. The difference between the study arms in the primary endpoint remained significant when adjusted for office SBP change (p < 0.001), office DBP change (p < 0.001), or pulse pressure change (p < 0.001). No significant interaction between changes in FMD and changes in HbA1c, SBP, DBP, or body weight was found. There was an increase and a decrease in the AUC for arterial diameter continuously measured during the first 5 min after cuff deflation for the dapagliflozin and glibenclamide arms, respectively (p = 0.001; Table 2).

**Table 2:** Absolute change in the endpoints after 12 weeks of therapy

	Dapagliflozin	Glibenclamide	p
Posttreatment change in the Rest FMD at 1 min, %	3.3 (8.2)	− 1.2 (7.5)	0.0001
Posttreatment change in the Post I/R FMD at 1 min, %	2.1 (9.4)	− 0.4 (11.1)	0.264
Posttreatment change in the AUC of arterial diameter during the 5-min course of Rest FMD, mm	0.5 ± 3.5	− 0.3 ± 3.5	0.001
Posttreatment change in the peak of systolic velocity, cm/sec			
At baseline	3.6 (39.9)	− 0.41 (29.8)	0.0001
At 1-min	6.7 (49.5)	− 2.3 (49.1)	0.0001
At 5-min	6.3 (32.3)	− 2.6 (47.0)	0.0001
Posttreatment change in the end diastolic velocity, cm/sec			
At baseline	− 0.13 (4.01)	− 0.12 (4.52)	0.387
At 1-min	− 0.02 (8.89)	− 1.15 (8.42)	0.001
At 5-min	0.77 (3.85)	− 0.51 (3.58)	0.0001
Posttreatment change in the resistive index			
At baseline	0.009 (0.06)	0.006 (0.07)	0.135
At 1-min	− 0.01 (0.12)	0.01 (0.10)	0.0001
At 5-min	− 0.02 (0.07)	0.01 (0.07)	0.0001
Posttreatment change in Nitrate levels during FMD, nmol/L			
At baseline	3.2 (16)	2.1 (10)	0.26
At 1-min	3.3 (17)	0.9 (18)	0.14
At 5-min	1.2 (10)	0.1 (14)	0.20
AUC during the 5-min course	5.6 (25)	2.2 (29)	0.15
Posttreatment change in Nitrite levels during FMD, nmol/L			
At baseline	0.013 (0.10)	0.019 (0.11)	0.135
At 1-min	0.031 (0.18)	0.009 (0.10)	0.0001
At 5-min	0.006 (0.07)	0.0013 (0.07)	0.0001
AUC during the 5-min course	0.18 (0.24)	0.01 (0.15)	0.0001

For the intragroup comparison of pretreatment and posttreatment rest FMD, there were significant differences during hyperemia (p < 0.0001) and at 1 min (p < 0.0001), 2 min (p = 0.001), 3 min (p = 0.007), 4 min (p = 0.015), and 5 min (p = 0.004) in the dapagliflozin arm (Fig. 1c). The same analysis identified no significant differences in the glibenclamide arm (hyperemia: p = 0.363; 1-min: p = 0.151; 2-min: p = 0.644; 3-min: p = 0.395; 4-min: p = 0.247; 5-min: p = 0.289; Fig. 1d). Baseline rest FMD at 1 min had a weak negative correlation with baseline HbA1c (r = − 0.15; p = 0.016). However, the change in 1-min FMD after 12 weeks of treatment was not associated with the change in HbA1c (r = 0.08; p = 0.13). No statistically significant interaction was found between prior coronary artery disease and the change in rest FMD at 1 min after the treatments (p = 0.45).

In the initial resting FMD, we observed paradoxical constriction 1 min after releasing the cuff in 31% and 29% of patients randomized to dapagliflozin or glibenclamide, respectively (p = 0.56). In this initial evaluation, absence of vasomotor response, *i.e.,* neither dilation nor constriction occurred in 1 patient from the dapagliflozin group. After 12 weeks of treatment, arterial constriction was no longer found in the dapagliflozin arm, but it remained present in 30% of those in the glibenclamide arm (p = 0.0001). In this final evaluation, absence of vasomotor response was observed in 4% of patients using dapagliflozin and in 30% of patients using glibenclamide (p = 0.0001). This endpoint was unplanned during trial design and must be considered exploratory.

Prespecified secondary parameters were analyzed to verify the effects of the treatments on microvascular resistance. The end diastolic velocity and the peak systolic velocity were both equivalent between groups at baseline (Table 1). The change in both velocities was distinct after 12 weeks of dapagliflozin compared to glibenclamide at both 1 and 5 min during the rest of the FMD test (Table 2). The resistive indices measured at baseline and at the 1-min and 5-min points were equivalent between the two study arms at randomization (Table 1). After 12 weeks of therapy, the change in the resistive index at 1 and 5 min during the rest FMD was significantly different between the dapagliflozin and glibenclamide groups (Table 2). No differences were observed between treatment arms in terms of anterograde or retrograde blood flow, anterograde or retrograde shear rate, or shear stress either at baseline or after 12 weeks of treatment (Additional 1: Table S2).

The change in post-I/R FMD dilation at 1 min increased a median of 2(9)% in the dapagliflozin arm and decreased a median of 0.4(11)% in the glibenclamide arm. Nevertheless, this difference did not reach statistical significance (p = 0.258) (Table 2). In accordance with the hierarchical statistical analysis strategy for the secondary parameters, we did not investigate the factors related to microvascular and macrovascular functions based on the diameter and velocity data from the post-I/R FMD.

### Plasma biomarkers of endothelial function

At randomization, no statistically significant differences between study arms were found in nitrite or nitrate levels at any of the three sampling times. After 12 weeks of treatment, there was a 10% increase in the 1-min nitrite level in the dapagliflozin group and a 4% decrease in the glibenclamide group. There were significant differences between study arms in terms of absolute value (p = 0.028) and the change from baseline to 1 min (p = 0.016). The AUCs for the three time points for nitrite level measurements were equivalent at baseline but increased by 24% (p = 0.004) after dapagliflozin and decreased by 14% after glibenclamide treatment (p = 0.24). No statistically significant changes were found in plasma nitrate or endothelin-1 levels between the study arms either at pretreatment or after 12 weeks of treatment (Additional file 1: Table S1).

### Plasma biomarkers of vascular inflammation and oxidative stress

No statistically significant differences were found between dapagliflozin and glibenclamide in terms of the pretreatment or posttreatment levels of systemic inflammatory activity markers (C-reactive protein, interleukin-2 (IL-2), interleukin-6 (IL-6), and tumor necrosis factor alpha (TNF-α), vascular inflammation markers (soluble vascular cell adhesion molecule-1 (sVCAM), soluble intercellular adhesion molecule-1 (sICAM), or systemic oxidative stress marker (free 8-isoprostane) (Additional file 1: Table S1).

### Adherence and safety

According to the intervention pill counts, the adherence rates at the first, second, and third months were 99 ± 3%, 97 ± 9%, 99 ± 4% in the dapagliflozin group and 98 ± 4%, 97 ± 6% and 98 ± 4% in the glibenclamide group. The alanine and aspartate aminotransferase levels did not differ between groups and did not change between randomization and week 12 (Additional file 1: Table S1). One adverse event was recorded in a patient in the glibenclamide arm who presented with transient acute confusion unrelated to hypoglycemia or clinical or radiological alterations characteristic of cerebral ischemia. A diagnostic hypothesis of progressive dementia was made, and the patient was referred for neurological investigation. The patient undertook the final study evaluation and was included in analyses predefined as intention-to-treat. However, the analyses performed after the patient’s data were excluded remained unchanged. Last, there were no adverse effects noted in the dapagliflozin arm.

## Discussion

The present study revealed that a 12-week course of dapagliflozin improves vasomotor function in the macro- and microcirculation of patients with T2DM and carotid atherosclerotic disease when compared to a 12-week glibenclamide course. Furthermore, this difference was observed in the setting of equivalent glycemia. Consistently, nitrite production during FMD increased after dapagliflozin treatment but not in the glibenclamide group. The coprimary endpoint, i.e., The change in FMD obtained after I/R was not different between the two therapies.

### SGLT2i and FMD

Three previous studies in T2DM reported inconsistent dapagliflozin effects on FMD [22–24]. The first study showed improvement in FMD after two days of treatment [23]; however, the authors reported no effect after 4 weeks of treatment [25]. In a second trial, although FMD was not significantly changed in the intention-to-treat analysis, a post hoc analysis indicated that an improvement was detected in a subgroup of patients with HbA1c > 7.0% at baseline. In the last study, 12-week treatment with dapagliflozin was compared with placebo, and although the change in FMD was inversely proportional to the change in HbA1c (r = − 0.4; p = 0.017), there was no significant improvement in FMD after dapagliflozin [24]. The main limitations of these trials were the selection of patients with FMD close to normal values as well as the suboptimal and unbalanced reduction in blood glucose in the study arms [24]. Hence, we carefully considered the equipoise of glycemic control and the presence of artery wall disease and dysfunction as requirements for designing the present trial. The pretreatment FMD values were ~ 1/2–1/3 of the mean values reported in the aforementioned studies [22, 23], suggesting that the patients in this study have worse vasomotor function than prior trials. The posttreatment HbA1c was 7.1% in both arms of the study. In light of this, our current study demonstrated that dapagliflozin leads to an improvement in FMD in individuals with severe arterial wall dysfunction when compared to glibenclamide as well as, in an intragroup exploratory analysis, when compared with baseline values. Notably, this difference occurred with equivalent glucose lowering between the two groups.

### Paradoxical arterial constriction

Approximately 30% of all patients included in the study had paradoxical arterial constriction after cuff deflation during the FMD protocol. This abnormal arterial response improved after treatment with dapagliflozin but not after glibenclamide treatment. We also observed a decrease in office systolic BP after dapagliflozin, but no difference in the measurements obtained at 24-h ABPM. Both findings suggest attenuation of the vasoconstrictor response to ischemic stimulation and of the white-coat effect after dapagliflozin [26]. The lack of effect of dapagliflozin on BP measured by 24-h ABPM is likely due to the careful clinical management performed in the run-in phase, which aimed to obtain groups that were comparable and suitable for the investigation of endothelial function. This strategy and finding strengthens the quality of the FMD analyses obtained in this trial.

Constriction of the brachial artery during the FMD protocol has already been described, as has its association with subclinical atherosclerotic disease [27]. There is scarce mechanistic information that was mostly obtained from studies with intra-arterial acetylcholine infusion, which has a strong correlation with FMD [28, 29]. In the normal functioning endothelium, acetylcholine promotes a rapid release of NO and, thus, vasodilation [30]. However, in the dysfunctional endothelium, the effect of acetylcholine that predominates is the contraction of smooth muscle cells causing vasoconstriction [30]. By inference, it is possible that the reduction in paradoxical vasoconstriction was favored by the improvement in endothelial function. Nevertheless, other nontested mechanisms may also contribute to this change, such as the direct action of dapagliflozin on vascular smooth muscle.

### Microvascular function assessment during FMD

To assess the entire artery wall response during the FMD protocol, we extended the capture period of the brachial artery diameter and flow velocity to 5 min after cuff deflation. In this manner, we intended to discern any prolonged effects of dapagliflozin on FMD, particularly on microvascular function. A prior study reported that blocking endothelial nitric oxide synthase (eNOS) with NG-monomethyl-*L*-arginine (L-NMMA) did not alter flow velocity at the peak of hyperemia but reduced the duration of hyperemia [31]. Our hypothesis was that if dapagliflozin acted on NO bioavailability, this effect could be better detected at the end of 5 min. In fact, in dapagliflozin-treated patients, we found a more pronounced change in both the peak systolic velocity and the end diastolic velocity between the study arms, mostly at 5 min after cuff deflation, suggesting a reduction in microvascular resistance. In fact, while in patients treated with dapagliflozin, there was a decline in the resistive index, in those treated with glibenclamide, this index increased.

As demonstrated in several studies [32–35], microvascular tone is mainly determined by hyperemic flow during FMD, which is particularly reflected in diastolic velocities during hyperemia [33]. As described above, the resistive index equation reflects resistance to blood flow, which can be greatly determined by microcirculation, particularly after cuff release. Thus, it is of special importance that, in this phase, our results show a significant difference. A similar result was reported in the resistive index measured in the renal artery following dapagliflozin treatment [23], corroborating this finding and indicating that this improvement in microvascular function may occur in multiple arterial beds. In the microcirculation, there is a nonlinear relationship between the diameter and the viscosity of the vessel, with the relative effective viscosity increasing six to seven times in vessels that are the size of capillaries [36]. The increase in blood viscosity shifts the curve of the association between the relative effective viscosity and diameter of the vessel upwards [37]. In the ADDENDA-BHS2 trial, blood viscosity increased as a result of treatment with dapagliflozin. Thus, the effect of SGLT2i in decreasing microvascular resistance is possibly underestimated by the concomitant increase in blood viscosity.

### Artery wall vasoactive molecules

Elevated plasma nitrite levels are indicative of increased NO production, and nitrate levels indicate the total available NO in the blood [38]. Dapagliflozin treatment increased nitrite production during FMD. In contrast, neither drug therapy altered nitrate levels. As commented above, SGLT2i activates eNOS and increases NO production [16], which may in part explain the improvement in both macrovascular and microvascular function [17]. Indeed, FMD is abolished after eNOS inhibition [31], and patients with impaired dilatory brachial artery responses do not present with any increase in nitrite levels [38].

The increase in the bioavailability of NO can also result from the reduction of its degradation, particularly by attenuating oxidative stress [39]. This possibility was considered in the study design based on a previous report indicating that dapagliflozin can mitigate oxidative stress as assessed by plasma 8-hydroxy-2′-deoxyguanosin, which is a biomarker of oxidative DNA damage [22]. We did not observe any change in the plasma levels of isoprostane, which is a marker of peroxidation of essential fatty acids. The contrasting results between these two studies may be due to differences in the two biomarkers of oxidative stress or differences in the studied populations. It is important to recognize that the lack of a significant change in isoprostane levels does not exclude the possibility of attenuation in oxidative stress and thus the reduction of NO degradation.

We found no significant difference in endothelin-1 levels between treatments. However, other mediators may have changed both the macrovascular and microvascular responses. Activation of the K_ATP_ channel is of particular relevance here, as glibenclamide was the active control group. Certain studies have shown that inhibition of K_ATP_ channels with sulfonylureas modestly attenuates hyperemia without affecting peak flow velocity [40]. Nevertheless, contradictory findings have been reported elsewhere [41]. We did not detect changes in nitrite level or systolic or diastolic flow velocity, but we found significant worsening of FMD at 1 min after glibenclamide treatment. In addition, the presence of arterial dilation during the FMD procedure decreased by almost half in the glibenclamide group and increased by almost a third in patients treated with dapagliflozin. This finding increased the differences between experimental arms and the significance of the primary outcome. However, the pretreatment vs. posttreatment analysis demonstrated that dapagliflozin was associated with significant increases in FMD at every time point throughout the 5 min of recording. Thus, dapagliflozin therapy had a direct effect on the measured endpoints.

### Post-I/R FMD

Endothelial dysfunction after I/R is a ubiquitous finding in several tissues and in a variety of species [42], and it has been associated with the extension of myocardial injury after an acute coronary event [43]. The reintroduction of molecular oxygen at reperfusion generates reactive oxygen species, which can directly inactivate NO. In line with this concept, we found that the levels of nitrate and nitrite both before and after FMD decreased substantially after the period of I/R in both study arms. Such attenuation of FMD after I/R undermined the statistical power for the difference between arms. Despite this, we noticed a slight increase and a minor decrease in post-I/R FMD in the dapagliflozin and glibenclamide arms, respectively, but these differences did not reach statistical significance. We speculate that the drop in FMD after I/R surpassed the endothelial improvement obtained with SGLT2i, particularly in individuals with established atherosclerotic disease. In agreement with that, no changes in oxidative stress, estimated by serum isoprostane, or vascular and systemic inflammatory markers were detected in either study arm. This experimental approach is unprecedented, and there is much room for clarification. For example, what is the expected range of the post-I/R FMD in the population with or without T2D, is it reversible and how long must the treatment time be to mitigate it. The present study was not able to exclude or confirm the SGLT2i effect on this endothelial function marker, but it generated data so that new studies can be planned with greater accuracy.

### Potential clinical implications

As mentioned above, current evidence and guidelines support the concept that SGLT2i can reduce the risk of myocardial infarction in individuals with prior manifestations of coronary events but not in those with uneventful clinical histories [4–6]. Clinical trials with follow-up long enough to verify the effect of SGLT2i on atherothrombotic events are unlikely to occur due to ethical limitations; i.e., the early reduction in hospitalizations for heart failure and cardiovascular mortality (1 month or less) [44] would interrupt the study before the effect of SGLT2i on arterial disease could be properly tested. This limitation is particularly important in individuals with a low annual incidence of cardiovascular events such as those in primary prevention. In this scenario, the present study is an alternative to indicate that among individuals in primary prevention, those with subclinical atherosclerotic disease may potentially benefit from SGLT2i from macro- and microvascular points of view.

### Limitations

The results of this study would be closer to the ideal if all enrolled patients had been using statins for a long period of time but statin effect on FMD reaches stability at long and although half of the patients did not use statins at admission, we did not include this therapy to avoid distortions in the magnitude of the effect of experimental treatments. However, statin use was balanced between the arms, and we found no interaction between the use of statins and the change in the primary outcome.

## Conclusion

The ADDENDA-BHS2 trial demonstrated that dapagliflozin treatment improves macro- and microvascular endothelial function in diabetic individuals presenting with atherosclerotic disease. This particular effect may be attributed to the fact that this agent increased NO production.

## Supplementary Information

**Additional file 1.** Supplemental material provides detailed information on the methods and exploratory analyses presented in the study.

## Data Availability

The principal investigators in this project and the BHS group held intellectual property rights for the research data. The datasets generated and/or analyzed during the current study are not publicly available, as proprietary work is ongoing. However, data may be made available by the corresponding author upon reasonable request.
