# Supplementary material for: Dapagliflozin effect on endothelial dysfunction in diabetic patients with atherosclerotic disease: a randomized active-controlled trial
Source: Cardiovasc Diabetol. 2021 Mar 26;20:74. doi: 10.1186/s12933-021-01264-z (PMC8004411; doi:10.1186/s12933-021-01264-z)
Supplement: Supplementary file 1 — Additional file 1. Supplemental material provides detailed information on the methods and exploratory analyses presented in the study. [file 12933_2021_1264_MOESM1_ESM.docx]

**Effect of dapagliflozin on endothelial dysfunction of diabetic patients with atherosclerotic disease: a randomised active-controlled trial**

Andrei C. Sposito^*1^, Ikaro Breder^1^, Alexandre A.S. Soares^1^, Sheila T. Kimura-Medorima^1^, Daniel B. Munhoz^1^, Riobaldo M.R. Cintra^1^, Isabella Bonilha^1^, Daniela C. Oliveira^1^, Jessica Cunha Breder^1^, Pamela Cavalcante_1_, Camila Moreira^1^, Filipe A. Moura^1^, Jose Carlos de Lima-Junior^1^, Helison R.P. do Carmo^1^, Joaquim Barreto^1^, Wilson Nadruz^1^, Luiz Sergio F. Carvalho^1^, and Thiago Quinaglia^1^, on behalf of ADDENDA-BHS2 trial investigators

1. State University of Campinas Medical School, Campinas, SP, Brazil

**Trial population**

Eligible patients were aged 40–70 y. Atherosclerotic disease was considered as: (i) carotid atherosclerotic disease defined according to standard guidelines and diagnosed by the presence of (1) carotid plaques, (2) carotid Intima-Media Thickness (cIMT) ≥ 1 mm, or (3) cIMT values > 75^th^ percentile for patient age, gender, and race based on their distribution in the Brazilian population, or (ii) coronary artery disease defined as previous myocardial infarction or coronary angiography showing ≥ 70% stenosis of ≥ one coronary artery. Other inclusion criteria were: (1) glycated haemoglobin (HbA1c) level between 7–9% and the use of ≤ two oral hypoglycaemic agents. Exclusion criteria were (i) glomerular filtration rates < 60 mL/min (calculated by the Chronic Kidney Disease Epidemiology Collaboration equation (CDK-EPI)); (ii) aspartate or alanine transaminase levels > 3× the upper reference limit; (iii) systolic BP (SBP) ≥ 140 mm Hg or diastolic BP (DBP) ≥ 90 mm Hg after 16 weeks antihypertensive medication adjustment during the run-in period; (iv) acute coronary syndrome, stroke, or coronary artery revascularisation within six months before enrolment; (v) plasma triglyceride levels > 500 mg/dL; (vi) polyuria, polydipsia, weight loss, or others clinical signs of volume depletion; (vii) insulin use; and (viii) pregnancy or reproductive age in female patients.

The study was planned evaluate the SGLT2i effect on FMD as compared with an equivalent glucose-lowering therapy. The choice of a sulfonylurea combined with metformin as a control group was based on the fact that this is the combination most often used in clinical practice(1). The dose of glibenclamide was selected to be equipotent to the maximum dose of dapagliflozin, *i.e.* 10 mg/day, in the blood glucose lowering effect.

**Procedures**

Eligible patients underwent an initial 16-week medication adjustment period. During this time, they were administered ≥0·5 g/day extended-release metformin and 25–100 mg/day losartan. Where a second hypoglycaemic agent was indicated, acarbose was administered at ≤ 300 mg/day to avoid the use of drugs with proven endothelial effects. Patients who remained eligible after the initial medication adjustment period were randomly assigned at a 1:1 ratio to dapagliflozin (10 mg/day) or glibenclamide (5 mg/day) treatment and stratified by gender, baseline HbA1c (7–7·9% or 8–9%), and body mass index (BMI) (< 30 kg/m^2^ or > 30 kg/m^2^). Administration of any additional glucose-lowering, lipid-lowering or BP-lowering agents was not permitted during the study timeline. Patients returned every 30 days for in-person follow-up evaluations of clinical and safety events and adherence to the study regimen until the twelfth week.

**Drug Dispensing, Adherence and Adverse Effects**

Study participants received a 30-day supply of their medications every 30 days. On delivery of the medicines, patients were instructed to bring used blisters on the next visit in order to calculate the percentage of the medication that was taken. Drug adherence was stimulated by telephone calls conducted by a team member every fortnight.

**Data management plan**

Data were recorded using REDCap electronic data capture tools hosted at Unicamp (2) and were stored into the database center of the University following the best practices and standards. Digital video data files generated in the FMD experiments have been processed and stored in AVI format and the same database center.

**Blood samples**

After 12-hour fasting period blood samples were obtained, centrifuged at 3500 rpm and thereupon measured for urea, creatinine, high-sensitive C-reactive protein (CRP), AST, ALT, glycemia, total cholesterol (TC), high-density lipoprotein cholesterol (HDL-C), and triglycerides (TG) (Cobas c702 Roche, Germany), insulin (Cobas e602 Roche, Germany), blood count (Sysmex XN-L-Series, US). Urinalysis and urinary creatinine/albumin ratio (Dimension RXL MAX, Siemens, Brazil) were performed on the same day. Glycated hemoglobin (HbA1c) was measured by high-performance liquid chromatography (HPLC) (D-100, Bio-rad, Brazil).

At the randomization and the 12-week visit, blood samples were also obtained, centrifuged and frozen at liquid nitrogen in order to measure: Interleukin 6 (IL-6), Interleukin 2 (IL-2), tumour necrosis fact alpha (TNF-alpha), Vascular Cell Adhesion Molecule 1 (VCAM-1), Intercellular Adhesion Molecule 1 (ICAM-1) (Invitrogen™ eBioscience™ ProcartaPlex Human VCAM-1, ICAM-1, IL-6, IL-2, TNF-alpha Simplex kit, USA), Endothelin 1 (ET-1) (ET-1 Human ELISA Kit Thermo Scientific, USA), isoprostane (8-Isoprostane ELISA Kit, Cayman Chemical Company, USA).

**Blood pressure**

Two clinic BP readings were taken at 60-second intervals after 3 minutes of rest using Omron HEM-705CP (Omron Healthcare, Japan) device, and their mean was defined as the office BP. The participant was then asked to stand up and an orthostatic reading was obtained after 3 minutes to assess for postural hypotension. A 24-hour AMBP was obtained within one week before randomization and up to one week after the 12-week treatment period (SpaceLabs, model 90207-8Q, USA).

**FMD analysis**

FMD was obtained after over-night fasting and after ten minutes of quiet resting in a supine position, and in a room with a controlled temperature around 22-25°C. As recommended by the Expert Consensus for the assessment of FMD (3), we systematically advised participants fast for at least six hours, avoid physical activity for at least 24 hours, do not ingest caffeine, vitamin C, polyphenols, alcohol and supplements that affect the cardiovascular system for at least 12 hours. The experimental treatments were administered, immediately before the FMD. The procedure lasted about 1 hour and soon after its completion the patients were fed.

The brachial artery was located above the antecubital fossa, and a longitudinal image of 6 to 8 cm of the artery was considered as the baseline scan. Video acquiring were blinded to treatment throughout the study and all analyses were performed after database lock, offline, and blinded to the patient treatment and study phase. A high-resolution ultrasound (Vivid S6; GE Medical System, Milwaukee, WI, USA) was used to measure brachial artery diameter. A probe holder (Quipu srl, Pisa, Italy) kept the ultrasound probe upright and allowed continuous recording of a 2D image and Doppler artery flow via a video capture device (DVI2USB 3·0^™^; Epiphan Video, Ottawa, ON, Canada) linked to a dedicated computer. The blood pressure cuff was placed below the imaged artery and was inflated to 50 mm Hg above systolic blood pressure for 5 minutes and then rapidly deflated. The total scan recording time was 11 minutes (baseline: 1 minute; ischaemia: 5 minutes; hyperaemia and dilation: 5 minutes). Automatic edge-detecting software (LabVIEW 6·02; National Instruments, Austin, TX, USA) assessed artery dilation and flow changes both online and offline. For the FMD, the percentage changes in arterial diameter and flow were calculated relative to the baseline scans. FMD assessments were performed at the time of randomisation and after 12 weeks dapagliflozin or glibenclamide treatment. Fifteen minutes after the first (rest) FMD, reperfusion injury of the brachial artery was created by cuff-induced ischaemia (50 mm Hg above the systolic blood pressure) for 15 minutes followed by 15 minutes reperfusion. The second (post I/R) FMD analysis was repeated and the aforementioned parameters were reassessed. The algorithm was set to detect leading edge flow, thus allowing us to determine the peak systolic velocity (PSV) and the end diastolic velocity (EDV). The PSV corresponds to each “peak” and EDV corresponds to the point marked at the end of the cardiac cycle, just before the next systolic. To mitigate artefacts or effects of turbulence, we carried out beat-to-beat data acquisition, increasing the signal/noise ratio and, during each offline analysis, an experienced analyser checked the entire route and when an artefact was detected, the section was excluded of analysis. The resistive index was calculated from the difference between the PSV and EDV divided by the PSV. The anterograde and retrograde shear rates were calculated as follows:

[4 × velocity-time integral / diameter] (1)

The anterograde and retrograde blood flows were obtained as follows:

[π × (diameter / 2)^2^ × velocity-time] (2)

The shear stress was also calculated as follows:

[(4 × η × velocity) / diameter] (3)

The shear stress established whether the SGLT2i treatment could possibly alter whole blood viscosity. The latter was calculated based on total plasma protein (TP; g/dL) and haematocrit (HCT; %) as follows:

[η = (0·12 × HCT) + 0·17 (TP - 2·07].(4) (4)

The consistency of FMD method has been improved by applying the currently recommended FMD technique(3) as well as good practices such as probe holders, forearm fixation, automatic detection of luminal edges, and offline blind analyses of examination recordings. Three experienced physicians (TQ, IB and STM) made the FMD procedures and the analysis were performed in a blinded fashion. In order to verify the accuracy of the study, we evaluated 25 exams performed by the 3 physicians and compared them with the software measurements. Inter observer coefficient of variability between the mean of the 3 physicians’ assessments and the software was 3.9%. The intersession coefficient of variability of the software for the same exam analysed by different observers in 10 different occasions was 0.26%.

Nitrite and Nitrate (NOx) levels in plasma were measured just before FMD and 1 and 5 minutes after brachial artery cuff deflation. This collection was repeated during the second FMD after ischemia and reperfusion. Samples were collected in a tube containing heparin, centrifuged at 3500 rpm in a refrigerated centrifuge and stored in liquid nitrogen in up to 3 minutes for further analysis of NOx by a NO chemiluminescence analyzer (Model NOA, Sievers Instruments, Boulder, CO, USA).

**Endpoints**

As previously described (5) the study was designed to determine whether there is a difference between the dapagliflozin and glibenclamide treatments in terms of the two primary endothelial function outcomes. The latter were defined as the difference between randomisation and 12 weeks of treatments in terms of the FMD obtained at 1 minute after release of 5-minute brachial artery occlusion at rest (outcome 1: change in rest FMD at 1 minute) and after a 15-minute ischaemia period followed by 15 minutes reperfusion (outcome 2: change in post I/R FMD at 1 minute). Prespecified secondary parameters included the area under the curve (AUC) of the diameter vs. time scatterplot and the derived parameters blood flow velocity, arterial diameter and shear rate, and plasma vascular and inflammatory marker activity levels.

**Statistical analysis**

The two study endpoints equally split an alpha level at the bilateral α=0·025. If one of them proved significant, the alpha value could then be recycled to test the secondary endpoints based on the index primary endpoint at the bilateral α=0·05. The study design projected that ~44 patients per arm would suffice to achieve β=90%. The sample size was increased to 49 per arm to guarantee this power level in the event that follow-up was lost and to provide power for the secondary endpoint analyses. Details of the sample calculation are published elsewhere(5) One patient in the study requested withdrawal of informed consent. Thus, analyses were performed on data from 49 patients in the glibenclamide arm and using data for 48 patients in the dapagliflozin arm. These analyses were conducted according to the intention-to-treat principle for testing superiority of SGLT2i over glibenclamide. Changes from baseline were compared between treatments for each primary endpoint. Data are means±standard deviation (SD) for normally distributed data and medians and interquartile ranges (IQR) for non-normally distributed data. Baseline continuous and categorical data were compared by Student’s *t* or Wilcoxon-Mann-Whitney *U* tests and a two-tailed Fisher’s exact test, respectively. Intragroup comparisons of pre-treatment vs. post-treatment values were performed by a related-samples Wilcoxon Signed Rank test or Student’s *t* test paired for variables with non-normal or normal distribution, respectively. Analyses were performed in SPSS v.22 for Macintosh (IBM Corp., Armonk, NY, USA).

Table S1. Laboratorial changes after treatments

|  | Dapagliflozin | Glibenclamide | p |
| --- | --- | --- | --- |
| Total cholesterol, mg/dL |  |  |  |
| At randomization | 164±40 | 165±43 | 0.655 |
| At 12-weeks | 170±42 | 165±41 | 0.914 |
| Low-density lipoprotein cholesterol, mg/dL |  |  |  |
| At randomization | 94±31 | 96±35 | 0.748 |
| At 12-weeks | 100±34 | 98±33 | 0.844 |
| High-density lipoprotein cholesterol, mg/dL |  |  |  |
| At randomization | 41±11 | 41±10 | 0.850 |
| At 12-weeks | 42±12 | 42±10 | 0.959 |
| Very Low-density lipoprotein cholesterol, mg/dL |  |  |  |
| At randomization | 29±15 | 28±10 | 0.686 |
| At 12-weeks | 29±20 | 26±10 | 0.435 |
| Triglycerides, mg/dL |  |  |  |
| At randomization | 187±119 | 178±94 | 0.674 |
| At 12-weeks | 202±214 | 161±79 | 0.217 |
| Alanine aminotransferase, mg/dL |  |  |  |
| At randomization | 30±16 | 29±19 | 0.745 |
| At 12-weeks | 29±36 | 28±16 | 0.822 |
| Aspartate aminotransferase, mg/dL |  |  |  |
| At randomization | 23±10 | 21±12 | 0.424 |
| At 12-weeks | 27±43 | 21±11 | 0.340 |
| Endothelin-1, pg/mL |  |  |  |
| At randomization | 0.30(0.34) | 0.24(0.35) | 0.652 |
| At 12-weeks | 0.26(0.12) | 0.25(0.12) | 0.662 |
| C-reactive protein, mg/L |  |  |  |
| At randomization | 1.9(2.2) | 2.2(4.1) | 0.210 |
| At 12-weeks | 1.7(2.8) | 1.7(3.4) | 0.546 |
| sICAM, ng/mL |  |  |  |
| At randomization | 395(280) | 420(225) | 0.795 |
| At 12-weeks | 404(264) | 387(206) | 0.624 |
| sVCAM, ng/mL |  |  |  |
| At randomization | 272(134) | 300(155) | 0.751 |
| At 12-weeks | 296(148) | 277(148) | 0.114 |
| IL-6, pg/mL |  |  |  |
| At randomization | 9.4(6.8) | 10.6(8.2) | 0.219 |
| At 12-weeks | 10(7.1) | 107.2 | 0.273 |
| TNF-α, pg/mL |  |  |  |
| At randomization | 11.7(6.9) | 11.9(8.2) | 0.631 |
| At 12-weeks | 11.0(6.3) | 11.9(6.6) | 0.795 |
| IL-2, pg/mL |  |  |  |
| At randomization | 67.0(37.7) | 59.8(39.0) | 0.725 |
| At 12-weeks | 71.0(51.1) | 64.9(40.1) | 0.927 |
| Free 8-Isoprostane, pg/mL |  |  |  |
| At randomization | 16.5(31.3) | 23.8(30.2) | 0.767 |
| At 12-weeks | 19.0(24.2) | 16.5(23.4) | 0.449 |

Table S2. Blood velocities and pressure during rest FMD

|  | Dapagliflozin | Glibenclamide | p |
| --- | --- | --- | --- |
| Anterograde Blood flow, mL‎/min |  |  |  |
| Baseline at randomization | 206(124) | 185(121) | 0.649 |
| 1-min at randomization | 178(176) | 171(154) | 0.977 |
| 5-min at randomization | 124(80) | 113(122) | 0.593 |
| AUC during FMD at randomization | 1285(809) | 1176(713) | 0.705 |
| Baseline at 12-weeks | 148(133) | 148(79) | 0.681 |
| 1-min at 12-weeks | 161(212) | 162(132) | 0.436 |
| 5-min at 12-weeks | 162(163) | 136(70) | 0.137 |
| AUC during FMD at 12-weeks | 1177(927) | 1143(609 | 0.149 |
| Retrograde Blood flow, mL‎/min |  |  |  |
| Baseline at randomization | -29(28) | -24(15) | 0.954 |
| 1-min at randomization | -26(21) | -25(15) | 0.535 |
| 5-min at randomization | -30(30) | -27(16) | 0.276 |
| AUC during FMD at randomization | 199(173) | 181(103) | 0.403 |
| Baseline at 12-weeks | -20(33) | -22(43) | 0.379 |
| 1-min at 12-weeks | -29(30) | -24(23) | 0.561 |
| 5-min at 12-weeks | -20(37) | -23(26) | 0.647 |
| AUC during FMD at randomization | 209(190) | 191(204) | 0.942 |
| Anterograde shear rate, s^-1^ |  |  |  |
| Baseline at randomization | 13.1(8.2) | 13.1(8.5) | 0.593 |
| 1-min at randomization | 11.3(12.1) | 11.0(9.6) | 0.960 |
| 5-min at randomization | 9.3(5.7) | 10.4(6.5) | 0.608 |
| AUC during FMD at randomization | 88.8(55.3) | 90.9(49.8) | 0.743 |
| Baseline at 12-weeks | 11.7(10.8) | 11.2(8.8) | 0.948 |
| 1-min at 12-weeks | 13.1(14.1) | 12.1(9.7) | 0.789 |
| 5-min at 12-weeks | 12.7(10.9) | 9.5(5.5) | 0.182 |
| AUC during FMD at 12-weeks | 92.2(61.6) | 87.0(49.2) | 0.624 |
| Retrograde shear rate, s^-1^ |  |  |  |
| Baseline at randomization | -1.9(2.2) | -2.1(1.0) | 0.948 |
| 1-min at randomization | -2.0(1.9) | -1.7(1.2) | 0.660 |
| 5-min at randomization | -2.4(1.4) | -2.3(1.5) | 0.773 |
| AUC during FMD at randomization | 15.6(11.9) | 13.8(7.6) | 0.616 |
| Baseline at 12-weeks | -1.5(2.2) | -1.8(2.1) | 0.223 |
| 1-min at 12-weeks | -1.7(2.0) | -1.9(2.2) | 0.274 |
| 5-min at 12-weeks | -1.4(2.4) | -2.1(1.8) | 0.282 |
| AUC during FMD at 12-weeks | 13.7(13.1) | 15.2(12.9) | 0.339 |
| Shear stress, dyn/cm^2^ |  |  |  |
| Baseline at randomization | 70(40) | 73(44) | 0.740 |
| 1-min at randomization | 60(65) | 59(61) | 0.977 |
| 5-min at randomization | 51(35) | 56(34) | 0.751 |
| AUC during FMD at randomization | 457(285) | 432(292) | 0.885 |
| Baseline at 12-weeks | 67(60) | 62(42) | 0.795 |
| 1-min at 12-weeks | 67(79) | 67(60) | 0.846 |
| 5-min at 12-weeks | 72(60) | 54(32) | 0.175 |
| AUC during FMD at 12-weeks | 493(304) | 434(235) | 0.525 |

**REFERENCES**

1. Margolis DJ, Leonard CE, Razzaghi H, Hoffstad OJ, Freeman CP, Nava KL, Molina T, Tan Y, Bartman BA: *Utilization of antidiabetic drugs among Medicare beneficiaries with diabetes, 2006-2009: Data Points #9*. Series DPP, Ed. Rockville (MD), Agency for Healthcare Research and Quality (US), 2012 Apr 5

2. Harris PA, Taylor R, Thielke R, Payne J, Gonzalez N, Conde JG: Research electronic data capture (REDCap)--a metadata-driven methodology and workflow process for providing translational research informatics support. J Biomed Inform 2009;42:377-381

3. Thijssen DHJ, Bruno RM, van Mil A, Holder SM, Faita F, Greyling A, Zock PL, Taddei S, Deanfield JE, Luscher T, Green DJ, Ghiadoni L: Expert consensus and evidence-based recommendations for the assessment of flow-mediated dilation in humans. European heart journal 2019;40:2534-2547

4. Nwose EU, Richards RS: Whole blood viscosity extrapolation formula: Note on appropriateness of units. N Am J Med Sci 2011;3:384-386

5. Cintra RMR, Soares AAS, Breder I, Munhoz DB, Barreto J, Kimura-Medorima ST, Cavalcante P, Zanchetta R, Breder JC, Moreira C, Virginio VW, Bonilha I, Lima-Junior JC, Coelho-Filho OR, Wolf VLW, Guerra-Junior G, Oliveira DC, Haeitmann R, Fernandes VHR, Nadruz W, Chaves FRP, Arieta CEL, Quinaglia T, Sposito AC, investigators A-Bt: Assessment of dapagliflozin effect on diabetic endothelial dysfunction of brachial artery (ADDENDA-BHS2 trial): rationale, design, and baseline characteristics of a randomized controlled trial. Diabetology & metabolic syndrome 2019;11:62

**Figure S1. Flow diagram of the study**

**
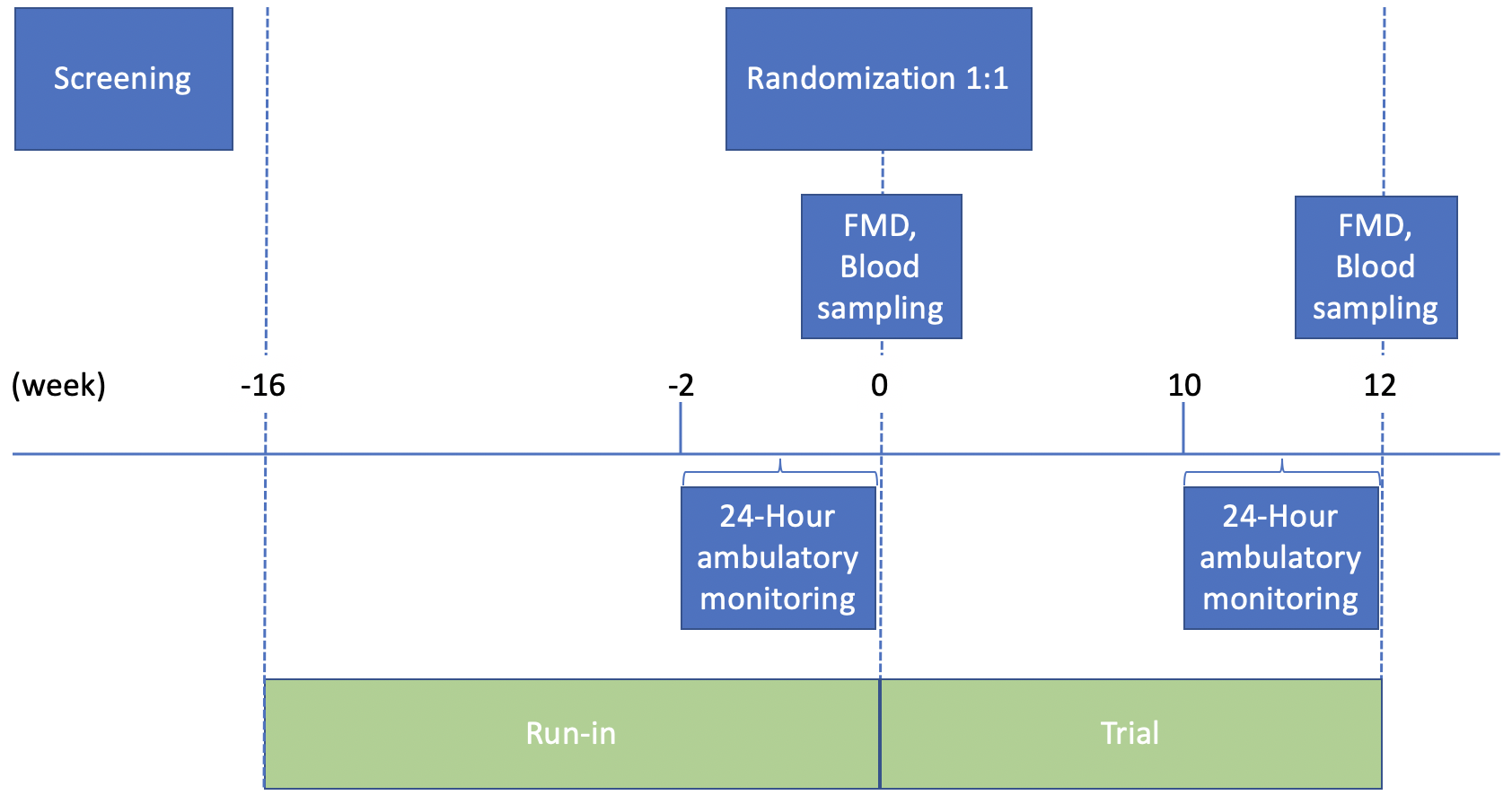
**

**Figure S2. CONSORT Flow Diagram**

**
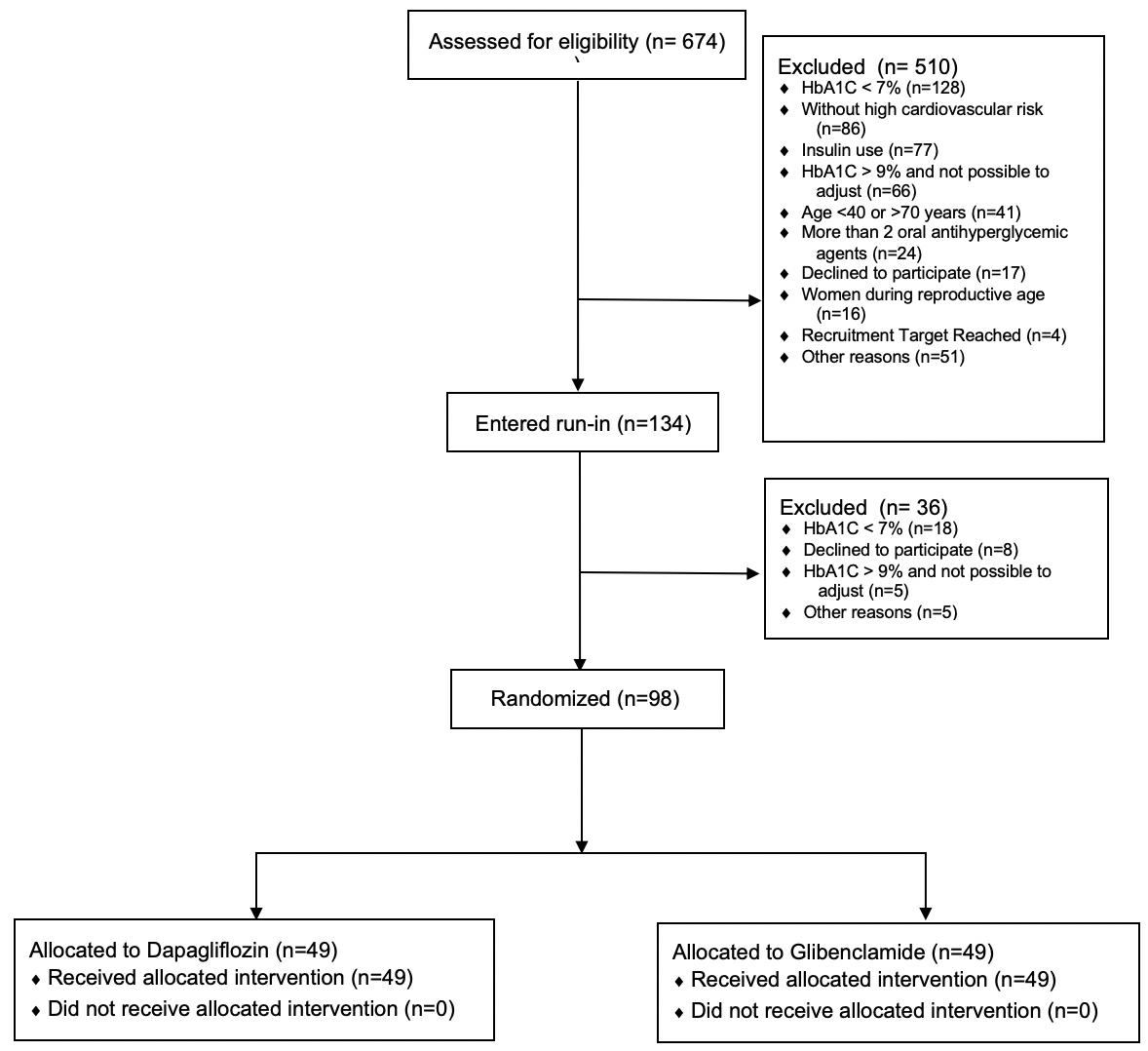
**
